# Landscape of HIV Implementation Research Funded by the National Institutes of Health: A Mapping Review of Project Abstracts

**DOI:** 10.1007/s10461-019-02764-6

**Published:** 2019-12-16

**Authors:** Justin D. Smith, Dennis H. Li, Lisa R. Hirschhorn, Carlos Gallo, Moira McNulty, Gregory Phillips, Michelle Birkett, Miriam Rafferty, Amrita Rao, Juan A. Villamar, Stefan Baral, Brian Mustanski, C. Hendricks Brown, Nanette D. Benbow

**Affiliations:** 1 Department of Psychiatry and Behavioral Sciences, Northwestern University Feinberg School of Medicine, Chicago, IL USA; 2 Department of Medical Social Sciences, Northwestern University Feinberg School of Medicine, Chicago, IL USA; 3 Institute for Sexual and Gender Minority Health and Wellbeing, Northwestern University, Chicago, IL USA; 4 Division of Internal Medicine, Department of Medicine, University of Chicago, Illinois, USA; 5 Shirley S. Ryan AbilityLab, Chicago, IL USA; 6 Department of Epidemiology, Johns Hopkins School of Public Health, Baltimore, MD USA; 7 Department of Preventive Medicine, Northwestern University Feinberg School of Medicine, Chicago, IL USA; 8 Center for Prevention Implementation Methodology for Drug Abuse and HIV, Northwestern University Feinberg School of Medicine, Chicago, IL USA

**Keywords:** Implementation research, Research funding, Mapping review

## Abstract

In 2019, the requisite biomedical and behavioral interventions to eliminate new HIV infections exist. “Ending the HIV Epidemic” now becomes primarily a challenge of will and implementation. This review maps the extent to which implementation research (IR) has been integrated into HIV research by reviewing the recent funding portfolio of the NIH. We searched NIH RePORTER for HIV and IR-related research projects funded from January 2013 to March 2018. The 4629 unique studies identified were screened using machine learning and manual methods. 216 abstracts met the eligibility criteria of HIV and IR. Key study characteristics were then abstracted. NIH currently funds HIV studies that are either formally IR (n = 109) or preparatory for IR (n = 107). Few (13%) projects mentioned a guiding implementation model, theory, or framework, and only 56% of all studies explicitly mentioned measuring an implementation outcome. Considering the study aims along an IR continuum, 18 (8%) studies examined barriers and facilitators, 43 (20%) developed implementation strategies, 46 (21%) piloted strategies, 73 (34%) tested a single strategy, and 35 (16%) compared strategies. A higher proportion of formal IR projects involved established interventions (e.g., integrated services) compared to newer interventions (e.g., pre-exposure prophylaxis). Prioritizing HIV-related IR in NIH and other federal funding opportunity announcements and expanded training in implementation science could have a substantial impact on ending the HIV pandemic. This review serves as a baseline by which to compare funding patterns and the sophistication of IR in HIV research over time.

**Electronic supplementary material:**

The online version of this article (10.1007/s10461-019-02764-6) contains supplementary material, which is available to authorized users.

## Introduction

HIV policy makers, providers, communities, and public health institutions have the evidence-based HIV interventions (EBIs) (e.g., antiretroviral therapies, pre-exposure prophylaxis (PrEP), self-administered HIV rapid testing) to achieve the goal of Ending the HIV Epidemic/Pandemic [1] world-wide [2–4]. Yet, in the US, overall HIV incidence has plateaued in recent years and is increasing among some populations [5]. AIDS remains the 6th leading cause of death among adults ages 25–44 [6]. A central challenge is clearly one of will and implementation.

The US National Institutes of Health (NIH) defines implementation research (IR) as “the scientific study of the use of strategies to adopt and integrate evidence-based health interventions into clinical and community settings in order to improve patient outcomes and benefit population health” [7]. Whereas traditional clinical efficacy and effectiveness research focuses on how a given practice affects health outcomes, IR focuses on how systems and implementation supports can be manipulated to improve intervention delivery [8]. Ultimately, IR aims to narrow the “chasm” [9] that exists between best-available research and routine practices in the real world by examining behaviors across the range of key stakeholders involved in delivery of EBIs and the contextual factors that influence successful adoption, implementation, and sustainment.

Accounting for 96% of all federally-funded HIV research investments in the US [10], the NIH is increasingly investing in IR to achieve a return on its investments in basic and clinical research. Initiatives include a growing number of funding opportunities and infrastructure support for IR, intra and extramural capacity building, and co-sponsorship of an annual meeting on dissemination and implementation science. Realizing the goal of eradicating new HIV infections will require leveraging the potential of IR more broadly and applying it to the unique challenges of HIV [11]. To date, documentation of the extent of IR integration into HIV research is limited.

We conducted a mapping review [12, 13] to assess the degree of recent investment in IR in the NIH’s HIV research portfolio. By capturing a snapshot of where IR has been funded for HIV interventions along the prevention and treatment continua, we aimed to characterize aspects of funded projects as they relate to IR aims and methods. Establishing this baseline can inform future study of trends and advancements in the intersection of IR and HIV and identify resource and training needs to ultimately eliminate new HIV infections across the US and globally.

## Methods

### Search Strategy

Using the publicly available NIH RePORTER (Expenditures and Results) module [14], we identified HIV-focused studies that received initial funding between January 2013 and March 2018 from NIH Institutes and Centers (search terms and limiters available in Supplementary Table S1). We included projects from all funding mechanisms except Small Business Innovation Research, Institutional Training, Construction Grants, Interagency Agreements, and Intramural Research, identifying 4629 unique studies based on award number.

### Screening and Eligibility

**Fig. 1:**
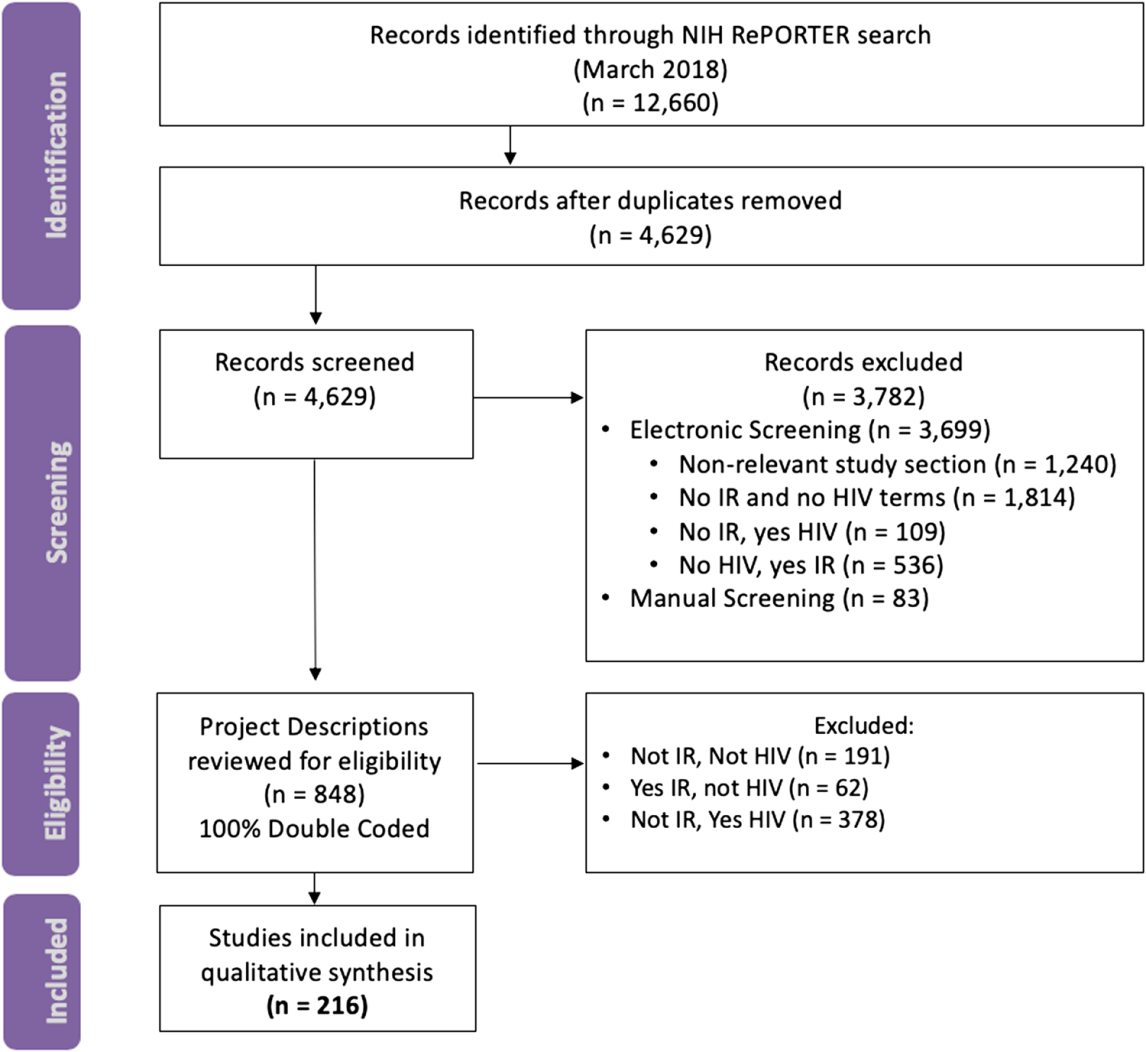
PRISMA flow diagram

We identified our study sample using a three-phase procedure (Fig. 1). Phase 1 comprised semi-automated computerized study exclusion. First, HIV basic science projects were automatically excluded based on the study section by which they were reviewed (n = 1240), as these sections would not review IR projects. Second, we used a text mining algorithm (with uncertain exclusions verified by human coding as noted in Fig. 1) to exclude studies based on the absence of any of 46 HIV and 31 implementation science terms in the project title and description, or the presence of any of 4 terms indicating the study was not yet at implementation phase (n = 2459 excluded). The terms were selected by expert opinion leaders in the fields of HIV prevention and treatment and implementation science (Supplementary Table S1). Third, we manually screened titles and project descriptions classified by the algorithm as nondefinitive, leading to the exclusion of 83 additional studies.

In Phase 2, the remaining 848 studies underwent a second review in which study authors double-coded each project description using Covidence [15] software. We had a 92% agreement rate on include/exclude decisions, with differences resolved by consensus. To be considered HIV, studies had to include an outcome related to the HIV prevention and care continua, PrEP, or HIV risk behavior. To be considered IR, studies had to evaluate the effect of implementation strategies, prepare for such an evaluation, or identify as a pragmatic or hybrid effectiveness–implementation trial; feasibility and efficacy/explanatory trials were excluded (Supplementary Table S2).

### Abstraction and Coding

The 243 studies that were coded as both HIV and IR underwent a full-text review and synthesis by the first and last authors. They independently coded each description and uncertainty was resolved via consensus leading to additional studies excluded that did not meet criteria. See Supplementary Table S3 for a bibliography of the final 216 projects.

We coded studies based on IR study characteristics and HIV intervention (definitions in Table 1). Based on the stated implementation aims, outcomes, and designs, we also categorized projects into one of five stages of an IR continuum developed for this review by the first author. Studies that did not evaluate the impact of implementation strategies (i.e., did not meet the NIH definition of IR) were classified as being in the “implementation preparation” (IP) phase, comprising three stages: (1) research only on barriers and facilitators; (2) studies to identify, select, develop, or adapt implementation strategies; and (3) pilot studies of implementation strategies (e.g., feasibility/acceptability). Projects meeting the NIH’s definition of IR (labeled as NIR) were categorized into two stages: (4) studies to evaluate the impact of an implementation strategy without a comparison or compared to implementation as usual; and (5) comparative implementation trials. We examined differences in funding, HIV intervention characteristics, and other IR characteristics by phase/stage along the IR continuum.

**Table 1:** Codebook

Variable	Coding/categories
HIV intervention characteristics	
Location	(1) United States, (2) international
Population^a^	(1) general population, (2) women, (3) adolescents/young adults, (4) men who have sex with men, (5) African Americans, (6) persons who inject drugs, (7) other substance users, (8) newborns/pediatrics, (9) incarcerated individuals, (10) sex workers, (11) transgender persons, (12) Latinx persons
HIV status	(1) HIV-positive, (2) HIV-negative, (3) both, (4) unspecified
Intervention^a^	(1) policy/law, (2) medical male circumcision, (3) couples counseling and testing, (4) sexual health promotion, (5) motivational interviewing/therapy, (6) HIV testing, (7) substance abuse treatment, (8) retention, (9) home HIV test kit, (10) combination HIV prevention, (11) integrated services, (12) behavioral intervention, (13) HIV care, (14) linkage, (15) ART initiation/adherence, (16) risk reduction, (17) PrEP, (18) HIV counseling/testing
HIV intervention continuum	Definition: single-intervention: primary prevention, secondary prevention, tertiary prevention/treatment. Multi-intervention. Split out by continuum
Implementation research characteristics	
Aim^a^	(1) adapt an EBI, (2) evaluate the impact of an adapted EBI, (3) understand barriers and facilitators to implementation, (4) select/develop/adapt implementation strategies, (5) evaluate the feasibility/acceptability of implementation strategies, (6) evaluate the impact of an implementation strategy, (6) compare implementation strategies, (7) unclear/other
Models^a^	Definition: used to describe and/or guide the process of translating research into practice; understand and/or explain what influences implementation outcomes (determinants); and evaluate implementation [16] Coding: open-ended
Strategies^a^	Definition: methods or techniques used to enhance the adoption, implementation, and sustainability of a clinical program or practice; actions taken on agents in the system of care itself and rarely only on the patient or client that is the recipient of the clinical program or practice Coding: open-ended
Outcomes^a^	Definitions available in Proctor et al. [17] (1) acceptability, (2) adoption, (3) appropriateness, (4) cost, (5) feasibility, (6) fidelity, (7) implementation, (8) process, (9) reach/penetration, (10) speed, (11) sustainability, (12) system effects, (13) not applicable, (14) none stated, (15) participant-level effectiveness
Design^a^	Adapted from Brown et al. [18] (1) developmental/formative/field/observational, (2) cohort/longitudinal/process, (3) within-site, (4) between-site, (5) within- and between-site, (6) modeling, (7) randomized trial with participant-level assignment
Stage of IR	Implementation preparation (IP): research in preparation for a formal evaluation or test, such as studies to understand implementation processes and barriers/facilitators; explore the feasibility or acceptability of novel strategies; development or tailoring of novel strategies; adapting an EBI; modeling that has potential to inform IR (1) identify barriers and facilitators, (2) identify/select/develop/adapt implementation strategies, (3) pilot implementation strategies NIH-defined implementation research (NIR): trials designed to evaluate the use of implementation strategies intended to integrate interventions into real-world settings; does not involve evaluation of clinical effectiveness outcomes (4) evaluate the impact of one set of strategies; (5) comparative implementation

^a^Not exclusive; can have more than one

## Results

### Funding Trends

**Fig. 2:**
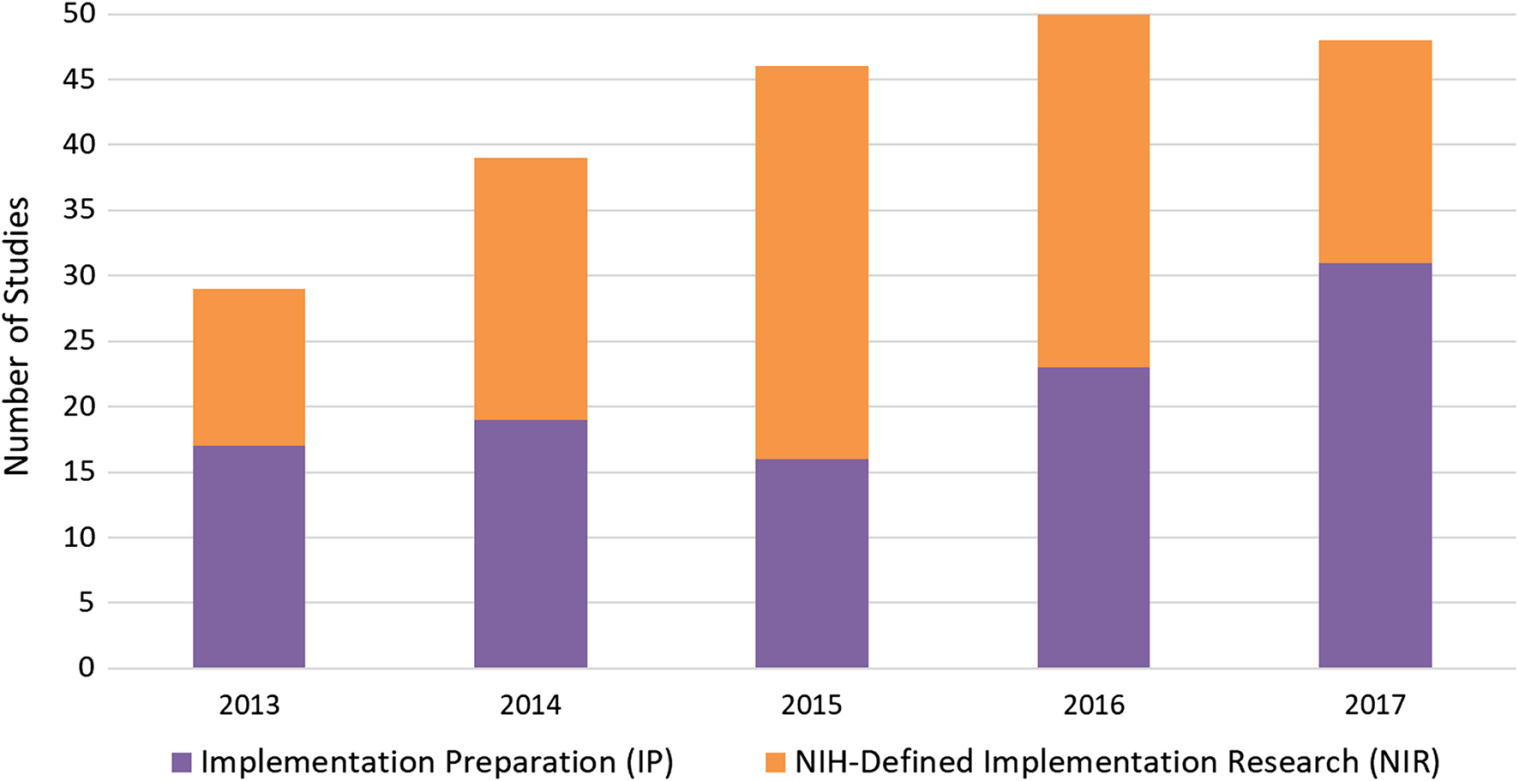
Number of studies by study start date and phase of implementation research

Studies were split between IP (n = 107, 49%) and NIR (n = 109, 51%). The number of funded studies increased from 2013 to 2016 and then saw a slight decrease in 2017 (Fig. 2); IP studies had a general upward linear trend over this period, while there were fewer NIR studies funded in 2017 than the previous 2 years. The majority of large-budget grants (R01/U-series) were NIR (n = 84) compared to IP (n = 33), whereas smaller-budget project codes were more likely to be IP (n = 63) than NIR (n = 18).

### IR Characteristics

#### Aims, Models, Strategies

The aims (n = 347, can be multiple per project) sought to evaluate the impact of implementation strategies (n = 94, 27%), understand barriers and facilitators to implementation (n = 73, 21%), select/develop/adapt strategies (n = 43, 12%); compare strategies (n = 37, 11%); evaluate feasibility/acceptability of strategies (n = 31, 9%); adapt an EBI (n = 29, 8%); and evaluate the impact of an adapted EBI (n = 17, 5%). The remaining IR aims (n = 23, 7%) were unclear or did not fit in one of the categories. Few (n = 28, 13%) project descriptions mentioned a guiding implementation model, theory, or framework. The most common were ADAPT-ITT [19], the Consolidated Framework for Implementation Research [20], RE-AIM [21] (n = 5, 2.3% each), and PRECEDE/PRECEDE–PROCEED [22] (n = 3, 1.5%); others were used 2 or fewer times. A total of 176 studies (82%) had at least one clearly defined implementation strategy. More than 150 different strategies were mentioned, with only a few being used in more than one study: adaptation of intervention/tailoring implementation strategies (n = 29), care coordination/integration (n = 14), incentivizing (n = 9), technology-based delivery (n = 6), community-focused (n = 5), home-based delivery (n = 4).

#### Outcomes and Design

In total, 290 implementation outcomes were mentioned, with more in NIR studies (n = 168) than IP (n = 122). Among the IP studies with implementation outcomes (n = 65, 61%), 23 (35%) mentioned one outcome, 32 (49%) two, and 10 (15%) three or more. For NIR studies with implementation outcomes (n = 81, 75%), 36 (44%) mentioned one outcome; 20 (25%) two; 25 (31%) three or more. Seven percent (n = 8) if IP and 18% (n = 19) of NIR projects mentioned only participant-level clinical outcomes. IP studies focused more often than NIR studies on evaluating formative metrics of implementation, such as acceptability and feasibility, whereas NIR studies were more likely to evaluate adoption, cost, reach/penetration, and sustainability (Table 2). Similarly, IP studies were more likely to use formative, observational, developmental, field, and modeling study designs, whereas NIR was more likely to use participant-level randomized controlled trials and between-site designs. Both used within-site; between- and within-site (e.g., stepped wedge); and cohort, longitudinal, and process designs at comparable rates.

**Table 2:** Comparison of implementation preparation (IP) and NIH-defined implementation research (NIR) projects on outcomes and designs

Implementation outcomes^a^	Implementation preparation (IP; n = 122) (%)	NIH-defined implementation research (NIR; n = 168) (%)
Acceptability	24	12
Adoption/uptake	3	5
Appropriateness	1	1
Cost	6	16
Feasibility	23	13
Fidelity	1	2
Implementation	3	4
Maintenance	2	4
Process	3	4
Reach/penetration	3	5
Scalability	–	< 1
Speed	–	< 1
Sustainability	–	7
System effects	–	< 1
Not applicable due to study aims/design	9	–
None stated when appropriate by study aims/design	10	4
Participant impact (effectiveness) only	13	21

Study design	Implementation Preparation (IP; n = 107) (%)	NIH-defined implementation research (NIR; n = 109) (%)
Developmental/formative/field/observational	50	–
Cohort/longitudinal/process	6	6
Within-site	4	7
Between-site	1	18
Within- and between-site	2	10
Modeling	12	–
Randomized trial with participant-level assignment	20	54
Not specified	7	5

^a^More than one outcome allowed per study

#### IR Continuum

We found 18 (8%) of studies examined barriers and facilitators, 43 (20%) developed implementation strategies, 46 (21%) piloted strategies, 73 (34%) tested a single strategy, and 35 (16%) compared strategies. Within the pilot trials, 28 (59%) included implementation outcomes. Among single-strategy trials, 38 (52%) included implementation outcomes, compared to 12 (34%) of comparative implementation trials.

### HIV Intervention Characteristics

Projects studied over 50 distinct target populations that represented combinations of age, sex, transmission risk, and other characteristics. Target populations of HIV interventions (more than 1 allowed per study) included general population (n = 25, 12%), women (n = 42, 20%), adolescents/young adults (n = 40, 19%), men who have sex with men (MSM) (n = 41, 19%), African Americans (n = 21, 10%), persons who inject drugs (PWID) (n = 20, 9%), other substance users (n = 21, 10%), newborns/pediatrics (n = 13, 6%), incarcerated individuals (n = 8, 4%), sex workers (n = 7, 3%), transgender persons (n = 5, 2%), and Latinx persons (n = 3, 1%). Target HIV status included HIV-positive (n = 67, 31%), HIV-negative (n = 58, 27%), both (n = 60, 28%), and unspecified (n = 31, 14%). Studies were split between international (n = 102, 48%) and US (n = 112, 52%) sites, with a greater percentage of international studies focusing on women (27% vs. 10%) and a greater percentage of US studies focusing on MSM (24% vs. 14%) and PWID (13% vs. 6%).

**Fig. 3:**
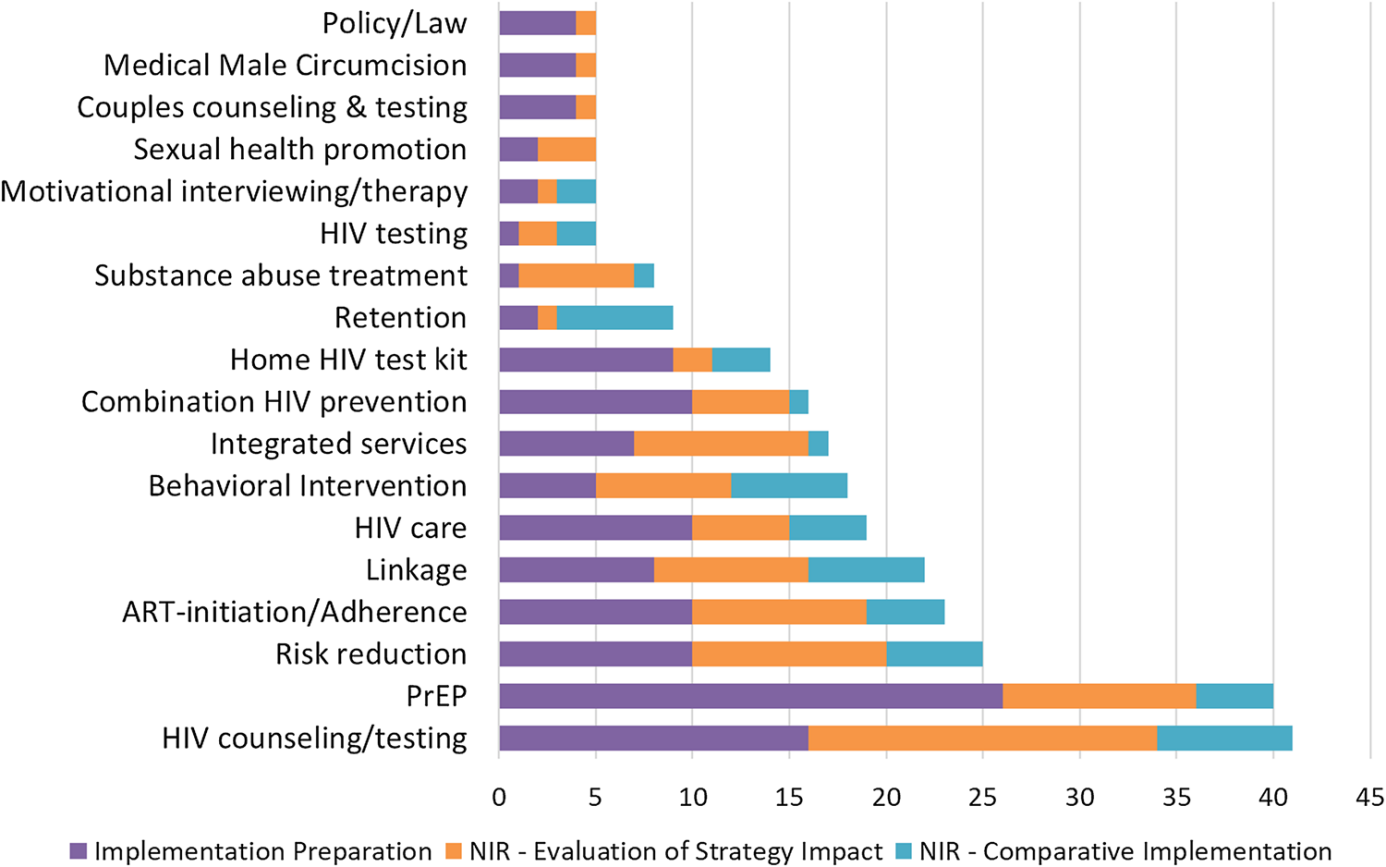
HIV intervention by phase/stage of implementation research. More than one HIV intervention allowed per study. *NIR* NIH-defined implementation research

Over one-half of studies (n = 123, 57%) focused solely on prevention with people who were HIV negative (e.g., PrEP, risk reduction) or on only one step in the HIV care continuum (e.g., linkage, retention, adherence). Of these, the majority targeted prevention (n = 74, 60%). Fewer targeted anti-retroviral therapy (ART) initiation/adherence (n = 23, 19%), HIV testing (n = 12, 10%), care retention and re-engagement (n = 12, 10%), and linkage to care (n = 1, 1%); 43% of studies were more complex, involving multiple prevention and/or HIV continuum steps. Most frequent among these were interventions that focused on retention-ART adherence (n = 18, 20%), testing-linkage (n = 14, 15%), prevention-testing (n = 8, 9%), prevention-testing-linkage (n = 7, 8%), or all continuum steps (n = 13, 14%). Substantial differences were noted between IP and NIR across HIV interventions (Fig. 3). Newer interventions (e.g., PrEP, combination prevention) had a larger proportion of studies in IP, whereas interventions with a longer history (e.g., retention, integrated services) had more studies in NIR.

## Discussion

The results of this mapping review indicate a small but growing portfolio of NIH-funded research that can contribute to understanding implementation of HIV interventions. On average there are four additional IR studies per year, and half of these, because they are in the IP phase, will likely produce limited generalizable implementation knowledge. However, advancing HIV IR goes beyond the amount of funding provided to the type, quality, and uniformity of the methods used. Our review revealed that NIH-funded HIV IR abstracts inconsistently used IR terminology and used methods more akin to efficacy or effectiveness research than IR. Given the potential growth of NIH’s portfolio of IR projects, we offer the following suggestions.

First, researchers can integrate best-practice IR methods into current studies so there is shared terminology and nomenclature to facilitate mutual understanding across the field. This includes using IR frameworks, models, and theories, which can be applied as the study is ongoing and upon completion [23]. More importantly, inclusion of, and emphasis on, IR outcomes are paramount to conducting IR studies in a meaningful way. It may seem contradictory to have included and classified studies as IR in this review if they did not include IR outcomes. However, we found numerous examples of studies with aims and designs commonly used in or unique to IR that only described patient-level clinical outcomes. We also identified some IR studies that only described one or two IR outcomes when many others would have been appropriate for the aims and design. Moreover, multiple IR outcomes are the norm in this field given their interrelated and multilevel nature [24, 25]. The limited use of implementation outcomes may reflect a difference between expectations of study sections for HIV research (e.g., more efficacy/effectiveness outcomes) compared to study sections for IR, which expect outcomes that address perspectives of multiple implementation stakeholders. Interestingly, there were more clearly identified implementation outcomes in IP than in NIR. This is perhaps due to researchers having more familiarity with outcomes like acceptability and feasibility, which were more prevalent in IP studies, than metrics like adoption, cost, reach/penetration, and sustainability, which were more common in IR studies.

Second, in order to measure most IR outcomes, researchers should consider aligning study designs with IR-related questions and aims—because the focus of implementation studies is the context that supports adoption, the designs and the outcomes used are different than those of a clinical trial [18]. IP projects were more likely to use formative, observational, developmental, field study, and modeling designs, while NIR projects were more likely to use participant-level randomized controlled trials and between-site designs. Even some studies further along the IR continuum (i.e., comparative implementation) used participant-level randomized designs more akin to a clinical comparative effectiveness study. This leads to a greater focus on patient-level outcomes and less on IR outcomes at the population or service delivery system level. These are missed opportunities to understand and quantify implementation on salient metrics at higher levels of the delivery system (e.g., providers, practices). Hybrid trials provide an opportunity for studying implementation within a predominantly effectiveness trial (type I) and for continuing to evaluate clinical effects even when implementation is the co-primary or primary aim (types II or III, respectively) and thus are optimal to ensure that studies address both implementation and effectiveness aims [26]. Many of the reviewed projects may actually have been hybrid trials without being labeled as such in the project description.

This review demonstrates that NIH funding in HIV prioritizes populations that align with the characteristics of the local epidemic where the studies are being implemented (i.e., MSM worldwide and PWID in US). Funding seems to follow the scientific needs of the field in that grant mechanisms with larger budgets and longer project periods were more likely to fund NIR, and studies of newer interventions were more likely to fall under IP. However, half of funded projects, including many involving interventions with evidence of effectiveness (e.g., HIV testing and linkage), still fell under IP. This could be attributed to the limited evidence for effective implementation strategies that need development and pilot testing. Nonetheless, there needs to be a funder- and researcher-driven shift toward conducting later-stage IR for these interventions to reach communities in need. This review may provide a basis by which to track that progression. Furthermore, NIH can use our findings to help increase the quality of reviews of IR in the field of HIV [27].

We also identified challenges that need to be addressed for HIV-related IR to mature. First, we noted a general lack of clarity around implementation strategies used in these projects, underscoring a need for distinguishing strategies from interventions [18]. Failing to make this distinction can lead to trial designs that preclude proper evaluation of IR outcomes. The naming of strategies will require a degree of standardization across projects, which is a field-wide challenge in IR not limited to HIV. Ongoing efforts are being made to organize and classify implementation strategies [28, 29], but ensuring that this nomenclature is adopted by HIV researchers inexperienced with IR will require its own set of dissemination efforts. Second, the observed decline in number of NIH defined IR studies in HIV in 2017 is concerning because the pandemic is ongoing. Increases in NIH funding can maximize return on investment of scientific discoveries and achieve their stated HIV priorities [30]. Such efforts are underway as part of the national *Ending the HIV Epidemic* initiative [1.]

## Limitations and Future Directions

The primary limitation was our available data source, namely that we reviewed project descriptions/abstracts from NIH RePORTER, which are brief and may contain insufficient detail of implementation aspects when they are not the primary aim of the project. Full proposals, specifically the Research Strategy section, would provide more detail but are not readily publicly available. We noticed a lack of uniformity in abstracts, which made it difficult to code some characteristics, and missing information, which could have resulted in under-inclusion and underestimation of IR in HIV despite our generous approach to coding IR characteristics. A future direction is to apply our coding system to published articles emanating from these studies. We also plan to validate our coding of project descriptions on a subsample of published study protocols and use machine learning to automate classifications for future studies. Furthermore, NIH-funded trials are just one source among many others where implementation of HIV interventions may be funded (e.g., PEPFAR, SAMHSA, HRSA, CDC, internally-funded pilot grants). Obtaining full protocols across the many funding agencies could be done via Freedom of Information Act requests, and investigators might be willing to fulfil such requests if assured that they would be presented in aggregate and thus unidentifiable. Last, descriptors did not exist for some of the coding we were interested in for this review; thus, categories were developed by the authors as a heuristic.

## Conclusions

Funded NIH research contributes to understanding the implementation of HIV interventions, but ending the epidemic could be accelerated with higher quality IR. Only a third of NIH HIV studies that have the capability to contribute to implementation knowledge are doing so, and of these just over half can be considered fully engaged in IR. There are already signs of investment from the NIH to further IR in HIV, as administrative supplements for the Centers for AIDS Research (CFARs) and AIDS Research Centers (ARCs) are available [31], and infrastructure support has been provided through the recent establishment of an Inter-CFAR Working Group on Implementation Science. NIH funding opportunities that influence IR in PEPFAR projects have continued [32]. The results of these investments are not yet known but can be tracked longitudinally via a similar review of newly funded studies in a few years. To move the needle, there is a pressing need for training to increase understanding of IR and the use of appropriate designs and outcomes for IR. Doing so, alongside funding opportunity announcements that are explicit about using IR best practices, will increase uniformity and comparability across studies. HIV has an arsenal of effective interventions. National and international efforts to get to zero new infections will lead to an infusion of more resources into this area of research and practice. With the tools available to end the epidemic, IR in HIV will be at the forefront.

## Electronic supplementary material

Below is the link to the electronic supplementary material.

Supplementary material 1 (DOCX 15 kb)

Supplementary material 2 (DOCX 21 kb)

Supplementary material 3 (XLSX 264 kb)
