# Supplementary material for: Landscape of HIV Implementation Research Funded by the National Institutes of Health: A Mapping Review of Project Abstracts
Source: AIDS Behav. 2019 Dec 16;24(6):1903–11. doi: 10.1007/s10461-019-02764-6 (PMC7220870; doi:10.1007/s10461-019-02764-6)
Supplement: Supplementary file 1 — Supplementary material 1 (DOCX 15 kb) [file 10461_2019_2764_MOESM1_ESM.docx]

**Exclusion Keywords**

1. Study sections identified as Basic Science:

AOIC

IHD

DDR

AIP

AMCB

NAED

1. Earlier intervention development terms:

Efficacy

Explanatory

Feasibility

Pilot

**Inclusion Keywords**

1. HIV domain terms:

Active referral

Adherence to ART

Adherence to antiretroviral therapy

Behavioral interventions

Biomedical interventions

Condom promotion

Condom use

HIV counseling

HIV testing

HIV screening

Targeted HIV testing

Linkage to Medical care

Linkage to Care

Data-to-Care

D2C

Re-engagement in Care

Medication adherence

Navigation services

Routine Opt-Out testing

Outreach

Partner services

Partner elicitation

Partner notification

Perinatal transmission

Mother-to-Child transmission

PrEP

Pre-exposure prophylaxis

Retention in care

Social marketing

Social network

Syringe services program

Needle exchange program

Syringe access

CLI

Community level interventions

Comprehensive risk counseling and services

CRCS

Group level interventions

GLIs

Health education

Risk reduction

HIV prevention counseling

Individual level interventions

ILIs

Prevention services

Structural intervention

1. Implementation related terms:

Acceptability

Adoption

Uptake

Utilization

Initial implementation

Intention to try

Appropriateness

Perceived fit

Relevance

Compatibility

Suitability

Practicability

Feasibility

Suitability for everyday use

Fidelity

Delivered as intended

Adherence

Integrity

Quality of program delivery

Reach

Penetration

Sustainment

Maintenance

Continuation

Durability

Incorporation

Integration

Institutionalization

Sustained use

Routinization

Diffusion
