# Supplementary material for: Landscape of HIV Implementation Research Funded by the National Institutes of Health: A Mapping Review of Project Abstracts
Source: AIDS Behav. 2019 Dec 16;24(6):1903–11. doi: 10.1007/s10461-019-02764-6 (PMC7220870; doi:10.1007/s10461-019-02764-6)
Supplement: Supplementary file 2 — Supplementary material 2 (DOCX 21 kb) [file 10461_2019_2764_MOESM2_ESM.docx]

**Supplementary Table S2**

Inclusion/Exclusion Criteria for Phase 2 - Double-Coding

1. Inclusion - Studies for which the answer is YES to BOTH to the items described below
2. Relevant HIV study

Studies that measure an HIV relevant outcome defined as:

- 1. measure an HIV relevant outcome related to the HIV care continuum indicators (e.g., knowledge of status, linkage to care, retention in care, on ART (adherence), and viral suppression)
  2. measure an outcome related to PrEP (awareness, linkage, retention, adherence)
  3. measure an outcome related to the reduction of HIV-related risk behavior.

1. Implementation study

- We define an implementation study as
  - A study that tests or evaluates the effects of an implementation strategy or multiple implementation strategies.
    - For example, include any abstract with a specific aim that says they are using an implementation science framework (e.g., RE-AIM) or implementation research design (e.g., stepped wedge implementation design; head to head comparison; pre-post; post; etc.).
  - A study that states use of a hybrid design that simultaneously evaluates both implementation and effectiveness in the same study
    - For example, any abstract that says they are a type 1, type 2, or type 3 effectiveness-implementation hybrid design. Joint focus looking at effectiveness of intervention and how it is implemented (e.g., process evaluation and/or documentation of implementation are minimum for Type 1).
  - A study that says they are looking at/exploring implementation barriers or facilitators.
  - A simulation, decision analysis, or statistical modeling study that varies factors affecting implementation delivery

1. Exclusion
   1. Studies for which the answer in A) was NO to at least one of the items
   2. Studies that only include: animal studies, basic research, device testing, medication dose treatment, type 1 clinical trials, or type 2 clinical trials
